# Implementation of early intensive behavioural intervention for children with autism in Switzerland

**DOI:** 10.1186/s12888-017-1195-4

**Published:** 2017-01-21

**Authors:** Nadja Studer, Ronnie Gundelfinger, Tanja Schenker, Hans-Christoph Steinhausen

**Affiliations:** 1 Department of Child and Adolescent Psychiatry, University of Zurich, Zurich, Switzerland; 2 Swiss Early Intervention Project in Autism, Swiss EIPA, Gland, Switzerland; 3 Clinical Psychology and Epidemiology, Department of Psychology, University of Basel, Basel, Switzerland; 4 Child and Adolescent Mental Health Centre, Capital Region Psychiatry, Copenhagen, Denmark

**Keywords:** Autism spectrum disorders, Health services, Intervention-psychosocial/behavioural, Pre-school children

## Abstract

**Background:**

There is a major gap between the US and most European countries regarding the implementation of early intensive behavioural intervention (EIBI) for children with autism. The present paper reports on the current status of EIBI in Switzerland and on the effectiveness of EIBI under clinical conditions in a Swiss pilot project.

**Methods:**

The paper combines a narrative report of the care system for children with autism in Switzerland and an initial evaluation of EIBI as implemented in the Department of Child and Adolescent Psychiatry, University of Zurich.

**Results:**

The current situation of the implementation of EIBI for children with autism in Switzerland is characterized by marked deficits in its acceptance. Major reasons include insufficient governmental approval and lacking legal and financial support. In addition, ignorance among health care providers and educational professionals has contributed to this situation precluding that children with autism receive the most beneficial assistance. The authors have initiated and been working in an intervention centre offering EIBI for a decade and report on their experience with the implementation of EIBI. Based on their clinical practice, they document that EIBI also works efficiently under ordinary mental health service conditions.

**Conclusions:**

EIBI needs to be implemented more intensively in Switzerland. Although the effects of EIBI as implemented in Zurich are promising, the results are not as pronounced as under controlled research conditions.

## Background

The first early intensive behavioural interventions in autism were implemented in the 1960s, when people with autism were still regarded as untreatable [1, 2]. The clinical psychologist Ole Ivaar Lovaas expanded on this work and other studies and developed a comprehensive intervention program for children with autism that was based on the principles of applied behaviour analysis (ABA) [3–6]. In his studies, Lovaas showed that the intervention was most effective when the treated children were younger than four years of age, when the parents were involved in the intervention, and when the intervention was very intensive (up to 40 h a week). Many modifications were made to early intensive behavioural interventions (EIBI) in the last 50 years. Punishment was eliminated, teaching became more naturalistic and the curriculum more comprehensive [7]. Models have also been developed to implement intervention in mainstream school settings [8, 9].

Various systematic reviews and meta-analyses of EIBI in children with autism have shown large effect sizes for cognitive functioning when compared with control data and active comparison interventions (e.g. [10–15]). There is also reasonable evidence for the effectiveness of EIBI compared to treatment as usual for young children with autism [16]. Even though these studies have demonstrated the effectiveness of EIBI under controlled research conditions, there are only few studies investigating the effectiveness of these interventions in clinical practice [8, 9, 17, 18]. These clinical studies have shown promising but less favourable outcomes. Furthermore, there is a remarkable gap in the implementation of ABA between North America and Europe as concluded in a recent review [19].

The purpose of the present paper is twofold, namely, (1) to provide a narrative review of the current situation in Switzerland in terms of diagnosis and treatment of autism in families with a young affected child and of the development and implementation of an ABA based early intervention program within a government subsidized clinic in Zurich, and (2) in terms of an evaluation to present some initial outcome data based on that model of practice. In contrast to previous studies, our findings were not predominantly obtained under controlled research conditions but rather in the real life circumstance of clinical service provision, and reflect the problems of implementing an innovative and highly structured and demanding intervention program into clinical practice. The methods of the evaluation part of the present paper will be described below following the detailed narrative review in the present background section. In this section, we will first focus on a general description of services provided to children with autism in Switzerland before turning to the description of the implementation of EIBI in Zurich.

### Autism in Switzerland

#### Services for children with autism in Switzerland

Health services in Switzerland are provided by public clinics and hospitals and by private doctors and hospitals. All 8 million inhabitants living in Switzerland are covered by a compulsory health insurance. Services for children with disabilities are organized within the public sector of the various cantons of the country. Screening for autism within routine paediatric check-ups in early childhood is not a standard procedure in Switzerland. Unfortunately, many paediatricians lack the expertise to detect the early signs of autism and often advise parents, who are concerned about the development of their child, to wait and see how the child develops. This can lead to a rather late diagnosis and intervention for children with autism. However, according to a recent parent survey [20], this delay has decreased in the recent past. Whereas the mean age at diagnosis had been 11.5 years for children born in the 1980s and 9.9 years for those born in the 1990s, it has gone down to 5.4 years for those born after 2000. Unfortunately, there are no valid prevalence estimates of autism for Switzerland.

There are very few specialised interventions available in Switzerland for children with an early diagnosis. Service delivery for children with autism depends heavily on where the family lives. Treatment for a preschool aged child typically consists of one hour a week of early special education. In a few regions, up to three hours a week of early special education intervention is granted. Furthermore, one or two hours a week of speech therapy and sometimes an additional hour of occupational therapy is offered in addition to the hour of special education. Most of the professionals providing these interventions have little to no experience working with children on the autism spectrum. Those professionals that do have experience usually cannot take in new clients because they have long waiting lists. According to the recent parent – based report [20] both preschool and school aged children with autism receive non- specific treatments like speech therapy and occupational therapy because these are treatments that are covered by the insurance companies.

In Switzerland, professionals providing EIBI are confronted with misconceptions and myths similar to those mentioned in recent reports describing the situation in other European countries [19] and the US [21]. ABA is not known as science, it is only known as a form of early intensive intervention for autistic children. ABA is still seen as equivalent to discrete trial teaching and not accepted by many professionals. Very few specialists working with young children with autism have training in ABA. Very little ABA is used in schools, most of the staff that are specialized in autism have training in TEACCH [22] and the so-called Affolter-model ®. The latter was developed in Switzerland and is based on the idea that autism is the result of a neuropsychological problem of perception. It is almost exclusively implemented in Switzerland. There is only one specialized school for autistic children in the German speaking part of Switzerland. This school does not use ABA either and does not have a Board Certified Behavior Analyst (BCBA) or Board Certified assistant Behavior Analyst (BCaBA) staff member or supervisor.

However, the picture exchange communication system (PECS; [23]), a behavioural intervention, is used by some special education teachers in classrooms and is sometimes used by early special educators and speech pathologists. There are only two certified PECS implementers in the country and both are not BCBAs nor are they non-certified behaviour analysts. The other professionals who have taken the basic PECS course do not have in depth knowledge of ABA either. Since many professionals using PECS in Switzerland have other theoretical backgrounds, they often do not use PECS the way a trained behaviour analyst would. PECS is often confused with TEACCH and is used to prompt the child’s receptive language skills or to talk about the child’s day. Systematic data collection is rarely carried out and the skills in systematic prompt fading are often insufficient so that many children are prompt dependent and are left without an effective form of communication.

As of February 2016, there are only eight BCBAs living in Switzerland and a small additional number living either in nearby Germany or France and working with families in Switzerland. Three of the Swiss BCBAs are working in the French and five in the German speaking part of Switzerland. Three of the BCBAs are working independently; two are working in a privately funded centre providing EIBI in the French speaking part of Switzerland and three are working in a government funded child and adolescent psychiatric service that is part of the University of Zurich. All of these experts are providing behavioural intervention for young children with autism.

In Switzerland, autism is declared a congenital defect and, therefore, medical treatments (psychotherapy, occupational therapy and physiotherapy) are not covered by the ordinary private health insurance but only by the public disability insurance. Since 2008, educational interventions (speech pathology, special education) are covered by the Swiss Conference of Cantonal Ministries of Education (EDK) and not by the ordinary health insurance or the invalidity insurance. Up until January 2014 the invalidity insurance did not accept EIBI as a scientific and appropriate medical method for the treatment of children diagnosed with autism. This was officially stated in two court decisions in 2004 and 2007 in which the request for financing EIBI was declined.

After ten years of pressuring the Federal Social Insurance Office, of which the invalidity insurance is part of, it was finally decided to at least partially contribute financially to EIBI for children with autism. The Office stated that EIBI is only partly a medical intervention and, thus, within the responsibility of the Office. The other part of the intervention is educational which lies in the responsibility of the ministries of education. The Federal Social Insurance Office now pays a flat rate for EIBI over two years. Children qualify for payment if they have a diagnosis of infantile autism, are under the age of 5, and receive at least 20 h of intervention a week run by one of six designated centres. The fee of 45’000 Swiss Francs (equivalent to 45 500 US$) set by the Federal Social Insurance Office is supposed to cover half of the intervention. Since early behavioural interventions are usually more intensive than 20 h a week this amount in fact only covers about a third of the costs of an intervention that lasts for two years with 35 h of intervention a week amounting to a total of 136 500 US$. The Federal Social Insurance Office sees the ministries of education being responsible for the other part of the costs.

The Federal Social Insurance Office selected six centres in Switzerland to provide early intensive intervention. Not all approaches are behavioural, two are non-behavioural. In two of the centres BCBAs are involved in the planning and implementation of the intensive intervention. In the other centres psychiatrists, clinical psychologists, special education teachers, language pathologists and occupational therapists are in charge of early intensive intervention.

In contrast to the Federal Social Insurance Office, which has the same guidelines for every canton, the ministries of education are working independently in the various cantons, deciding which kind of educational intervention and how many hours of treatment are paid for in each canton. Even though two cantons had indicated their willingness to cover their share of treatment, so far, only one of the approached cantons has financially contributed to EIBI. The other cantons either see legal obstacles in the way of contributing by indicating that when the intervention is run by a psychologists it is considered psychotherapy which is a medical and not an educational intervention so that they are legally prohibited to pay for services; or they argue that their support for families with a child diagnosed with autism is already sufficient. This leaves all the families living in the other cantons paying for over half of the expenses for early intensive intervention. The service providers try to assist the families in finding supportive associations that pay for at least part of the expenses. Since not every family gets a treatment place in one of the designated centres, parents have to look for a private EIBI provider and are left with paying the full cost of the intervention. In conclusion, despite many years of fighting for an adequate reimbursement of the high costs of EIBI and despite various court initiatives, the current situation of financing early intervention is still very unsatisfactory.

#### Early intensive behavioural intervention in Zurich

For more than 90 years, the Child and Adolescent Psychiatric Service (CAPS) of the canton of Zurich has served the needs of children and adolescents with mental disorders. The service is financially subsidized by the government of the canton and contributes to teaching and research as a Department of Child and Adolescent Psychiatry, University of Zurich. The institution has some 400 employees offering outpatient, day-clinic, and inpatient services for the entire canton of Zurich. This year, the CAPS merged with the Department of Adult Psychiatry by forming the Department of Psychiatry, University of Zurich.

Based on joint initiatives by an American father of a child with autism living in the canton of Zurich and the director of the CAPS, EIBI was initiated in Zurich in 2004. It soon became necessary to expand the program. Therefore two psychologists were sent for a year-long training to the Lovaas Institute in New Jersey in order to become EIBI supervisors.

After returning from training in the USA, the psychologists started the EIBI program in Zurich by translating treatment guidelines into German and adapting the program to the local needs. Since minimum salaries are rather high in Switzerland and intervention costs had to be kept affordable, the program was set up in collaboration with the University of Zurich, starting a University based internship for undergraduate psychology students to work as co-therapists. Unlike other internships, this was a part time internship spread out over a longer period of time. Co-therapists worked two to three sessions a week with one child for at least one year.

Furthermore, the two psychologists started teaching a University course on EIBI within the masters program of clinical psychology. Close supervision of the EIBI program was performed by Linda Wright, the clinical director of the Lovaas Institute, twice a year for a week and with a frequent exchange of e-mails.

The CAPS offered two intervention models that were implemented in the families’ home: one for families that lived near the clinic with intensive supervision and one for families that lived further away (more than an hour from the clinic) with remote supervision. For the intensive supervision model, the intervention team consisted of co-therapists employed by the CAPS. There was a two-hour team meeting every other week, where bigger adaptations to the program were discussed and demonstrated. Smaller adaptations could be made by the more experienced co-therapists throughout the week. The children received up to 30–35 h a week which averaged at about 25–30 h due to illness, vacation, therapist turnover and other unforeseen losses. Most of this time was spent at a rate of one child and one co-therapist. In the course of the two years of intervention, some of the children (7/23) started kindergarten. The intervention model for those kids stayed the same if the number of intervention hours a week were 15 or more.

The parents were involved in the intervention and were full team members and co-therapists for at least the first 6 months of intervention. After that, the focus usually changed to everyday life goals. But both parents still were required to be part of the intervention and to learn to teach their child effectively. Unfortunately, the parents were often not as involved in the intervention as we would have wanted them to be. This had an impact on generalizing treatment goals to everyday life and maintaining skills.

In the remote supervision model, the parents hired their own co-therapists and the team met about once every four weeks for a four-hour team meeting, which included the training of the co-therapists. The parents were free to choose how many hours of intervention they wanted to do and how involved they wanted to be. It was often difficult for families to find affordable co-therapists to get up to 35 h a week. Treatment progress was monitored closely and a more extensive evaluation in terms of quality control was made after the first four children had finished two years of intervention. Many changes were made to the intervention by realizing that the team had relied too much on discrete trial teaching and had neglected incidental or natural environment teaching. Instead of starting by working on compliance and discrete trial teaching we started to teach requesting first and spent more time on pairing. Parents were also involved more in the program and there was a greater focus on teaching in a more generative way.

With little support and possibility to connect with other behaviour analysts in Switzerland, the urge for further education was strong. Thus, Eric Larsson, executive director of the Lovaas Institute Midwest, was hired as a second supervisor. Supervision frequencies were changed to an annual visit and Skype supervision every other week. Four of the Zurich psychologists took the coursework to become Board Certified Behaviour Analysts, two of them took the exam and are now BCBAs. The other two meet all the requirements to take the certification exam but have not taken it yet. At the end of 2015 a third BCBA who had learned and trained in Ireland and Germany could be hired.

One of the major obstacles we faced was the recruitment and the training of the co-therapists. Because the salaries are not competitive, we have very dedicated and reliable staff but also a large turnover. New staff has to be trained every couple of months, which takes up a large part of the available supervision time. With staff only working a few hours a week, training new co-therapists took a very long time. In 2008, we started a more standardized training that initially was very similar to the training provided at the Lovaas Institute in the USA. Various adaptations were made over the years to make it more suitable for our needs.

Currently, we have a three-level training. The first and basic level consists of 23 h of lectures and about 8–10 h of lecture and role-play given by the supervisor in form of workshops at the beginning of working with a client. It is completed when a co-therapist has worked 250 h of 1:1 with a child, has taken part in regular team meetings, has learned to write reports, has completed all the lectures, and has passed a written and practical exam. The co-therapist then becomes an experienced therapist with a slight raise in salary and more responsibility and training possibilities within their working team. Many of the co-therapists then move on to working with another client. The experienced therapist further completes 23 h of lectures, gets trained in training new co-therapists and helps with the assessment and analysis of behaviour. After completing requirements and passing a written and practical exam, this advanced level of training is completed.

Ideally every client’s intervention team has one senior co-therapist. This co-therapist has passed the advanced level training and helps with training the team of co-therapists and stakeholders under the supervision of the supervisor. The senior therapist has the possibility to participate in an assistant supervisor course, which consists of 60–70 h coursework plus reading assignments, learning to use different assessment tools, and learning to write long and short-term goals for individual learners. The assistant supervisor course is completed after a written and a practical exam have been passed. The position of an assistant supervisor can then be pursued. Because it has been difficult to recruit enough senior therapists and assistant supervisors, not every team had these more skilled therapists.

The basic level courses are led by experienced or senior therapists under the supervision of the supervisors. The advanced level courses are led by senior therapists and assistant supervisors under the supervision of a supervisor. The assistant supervisor courses are led by supervisors. All three training levels include on-the-job training during 5-10% of the total hours spent working with the client. This standardized training increased the quality of training including treatment fidelity immensely, but with most of the co-therapists still working for only two sessions a week, the complete training takes them a long time and since salaries are not competitive the turnover is still high and most of the co-therapists leave after the first level of training.

After the Federal Social Security Office introduced the flat rate for early intervention, the supervision model for EIBI had to undergo further changes. The remote supervision model had to be dropped and the number of hours of supervision had to be decreased. Thus we could no longer provide the high level of supervision for the co-therapists which is essential to the high quality of the intervention. A new position was introduced, namely, the assistant supervisor. This person runs the treatment program under the close supervision of the supervisor and especially does a lot of the staff training. Unfortunately, the flat rate only covers a large part of the cost of the supervisor and the families are left paying for the assistant supervisor in addition to the other co-therapists.

The supervisor oversees the program planning, training of the co-therapists and stakeholders, and meets with the parents for monthly meetings. The assistant supervisor meets with the supervisor every week to discuss the child’s specific goals, progress, problems and training of the team. Since its inception, the EIBI program at the CAPS has trained and supervised more than 200 co-therapists. The introduction of the flat rate brought many restrictions to our program like having a minimum number of hours of intervention a week and limiting availability of intervention to children diagnosed with infantile autism (and excluding children with atypical autism).

In sum, the Zurich model of EIBI is comparable to similar models implemented in other parts of the world with the same common features [24]. These include a comprehensive and individualized treatment model, the use of behaviour analytic procedures to build functional repertoires and to reduce interfering behaviour, and the provision of one or more co-therapists with advanced training in ABA and experience with young children with autism directed treatment. Furthermore, the intervention follows typical developmental sequences when selecting treatment goals and short-term objectives, and expects the parents to actively participate as co-therapists in their child’s intervention. The delivery of treatment starts in a one-to-one fashion and has a gradual transitioning into small-group and large-group formats when warranted. In addition, the treatment starts in the child’s home and carries the skills over into other environments with gradual, systematic transitions into kindergarten. Finally, the intensive intervention includes 20–35 h a week with a duration of two years or more and starts in the preschool years.

## Evaluation of the Zurich EIBI project

### Methods

Parallel to the installation of the EIBI program in Zurich, an attempt was made to monitor the effects of the intervention in each individual child by use of a battery of assessment tools. The primary aim was to run a clinical quality control because it would have been premature to perform a highly demanding randomized controlled trial. The following description is therefore based on the retrospective analysis of data extracted from the individual files by the main responsible authors of the present contribution (NS and HCS). The evaluation does not represent a clinical trial. It was our aim to perform this pilot study to guide our registered prospective trial which has been started in 2014 and will be delivering results only in some years. It was expected that this pilot intervention would result in measurable changes of autistic features and developmental progress.

### Measures

The battery of repeated assessments contained the Autism Diagnostic Observation Schedule (ADOS-G, German version by [25]), cognitive testing (Snijders-Oomen non-verbal intelligence test; SON-R 2 ½ - 7, SON-R 5–17 by [26]), adaptive functioning (German short Version of the Vineland Adaptive Behaviour Scale, VABS (Rühl D: Vineland-Rating Scales, Unpublished paper) [27] and the Parenting Stress Index Short Form (PSI; [28]). A psychologist experienced in testing autistic children working in the CAPS but independent of the early intensive intervention program tested the children before the start of the intervention and then annually. The ADOS-G was administered by a psychiatrist with 20 years of special expertise in autism and trained to clinical reliability in the use of the ADOS-G. Testing adaptations had to be made especially for intelligence testing. The suggestions by Freeman [29] were followed so that the test was broken down into smaller segments and compliance rather than correct answers was reinforced.

### Sample

Between April 2004 and March 2014, 23 children (19 boys and 4 girls) had been treated by the CAPS Zurich and had finished at least two years of intervention. Inclusion criteria were the following: age less than five years, diagnosis of autism according to ICD-10 criteria (F.84.0), residence that could be reached within an hour (except for the remote supervision treatment model), adequate command of German or English language, sufficient capacity of the parents to cooperate with the rather demanding treatment prerequisites, and availability of treatment resources. One boy left treatment after one year. The average age at onset of intervention was 45.04 months (range 28–59 months). The children were diagnosed according to ICD-10 criteria. A total of 22 children had a diagnosis of infantile autism and one child had a diagnosis of atypical autism.

One child was excluded from the sample because he technically did not fit to the inclusion criteria for the intervention. He was just turning six when we started with the intervention and it was not comparable to the intervention of the other children with more hours in a school setting and school staff not trained or supervised by the early intervention supervisor. One other child had been treated in the remote model of intervention but his data has not been collected systematically. In the beginning of the project, systematic data collection was only intended for children receiving the intensive model of intervention. Unfortunately, data on the number of children who received an initial evaluation but did not enter into treatment was not collected systematically.

Three of the 23 children had one additional diagnosis (Q92: other trisomies and partial trisomies of the autosomes, not elsewhere classified, Q37.1: cleft hard palate with unilateral cleft lip, F90.1: hyperkinetic conduct disorder), two children had two additional diagnosis (Q67.4: other congenital deformities of skull, face and jaw and H50.0: convergent concomitant strabismus, E34.2: Ectopic hormone secretion, not elsewhere specified and L20: atopic dermatitis) and one child had four additional diagnosis (Q04.9: congenital malformation of brain, unspecified, Q04.3: other reduction deformities of brain, G40.2: localization-related (focal) (partial) symptomatic epilepsy and epileptic syndromes with complex partial seizures and G80.4: ataxic cerebral palsy).

Only four of the children enrolled in the program had a Swiss mother and father, nine children had one Swiss parent (7 mother, 2 father) and ten children had two non-Swiss parents (4 from Europe, 6 from outside Europe). Before the children were enrolled in the intervention program, 14 families were speaking a different language or an additional language than they ended up speaking when they started the intervention. The intervention language was either (Swiss-) German or English (only one child). Only three out of the 23 children were treated with the remote supervision model. This number was deemed too small for any useful analysis comparing the intensive vs. remote models of intervention.

### Data analysis

Because the main focus of the EIBI project was the treatment of the children rather than the evaluation of our intervention model, there were, unfortunately, missing data. For the IQ measurement, we had the problem of testability at baseline which is common with severely disabled children. Even though we used the non-verbal SON-R test and we made the above mentioned adaptations to test administration, only six of the 23 children could be tested for their IQ at baseline. However, after one year of intervention 20 out of 23 children were testable for IQ and after two years of intervention all children were testable for IQ. Both the SON-R developmental total and the SON-R total standard score IQ were used for analysis.

The VABS screener was used to get more information from the parents about the four domains of communication, socialisation, daily living and motor skills. There were missing data too. Since a clinical protocol for administration, which does not allow the calculation of standard scores, was followed, only a developmental age level (months) for each domain was obtained. In addition, a developmental age (DA) quotient was calculated for each child by dividing the child’s developmental age by the chronological age and multiplying it by 100, as performed in the study by Flanagan et al. [17]. The VABS evaluates motor skills only up until the age of six. Thus, motor skills for children aged above six cannot be analysed. This was relevant especially after two years of intervention because nine children were older than six years at this time.

We faced further difficulties with the analysis of the ADOS-G. A different module comes into use when the language skills of the children increase. Five children started off with Module 1 for children with no language to few words and were tested with Module 2 after two years of intervention.

Due to missing VABS and ADOS data, a comparison of the ADOS and VABS scores of children that could be assessed for IQ at baseline and those that were not testable could not be administered. Only one out of the six children had a complete set of data. Four of the six children were assessable on the ADOS Module 2 after intervention so that the ADOS scores cannot be compared. The VABS data was also incomplete for the six children. Four children had data at baseline and after two years of intervention and only two children also had data after one year of intervention.

Data analysis compared mean scores at baseline and after one and two years of intervention, respectively, except for the ADOS-G, which was assessed only at baseline and after two years. Considering the non-normal distribution of scores, non-parametric Wilcoxon tests were used for all comparisons of the data. In addition, effect sizes *r* were calculated. The effect sizes can be interpreted as follows: .10 -.29 = small, .30 -.49 = medium, and ≥ .50 = large.

## Results

The results of the evaluation are shown in tables 1 and 2. It should be noted that the samples shown in these tables differ in size but are partly overlapping. After one year of intervention, significant gains were obtained in the domains of communication skills DA and DA quotient daily living skills DA and motor skills. The effect sizes were all large (table 1). As expected and reflected by the number of significant differences and the effect sizes, the most significant positive changes were seen after two years of intervention. Children in the program displayed significant changes in all VABS domains except for the developmental age quotient of daily living skills and motor skills. Again, all effect sizes were large (table 2).

**Table 1:** Outcomes for children at baseline and after one year of treatment

		Baseline		After one year			
Measures	n	M	SD	M	SD	Z	*r*
VABS communication skills DA	13	9.7	8.8	15.9	12.3	−2.870^*^	0.80
VABS communication skills DA quotient	13	19.7	12.2	27.8	18.8	−2.412^*^	0.67
VABS daily living skills DA	13	18.7	5.4	24.3	8.3	−2.550^*^	0.70
VABS daily living skills DA quotient	13	45.3	11.5	44.4	11.5	−0.715	0.20
VABS socialisation skills DA	13	10	5.8	14.7	11.8	−2.004	0.56
VABS socialisation skills DA quotient	13	23.6	12.6	26	17.6	−0.245	0.07
VABS motor skills DA	13	25.7	8.5	36.7	13.9	−2.764^**^	0.76
VABS motor skills DA quotient	13	62.3	18.2	66	20	−0.874	0.24

*p < .05; **p < .01; ***p < .001

*DA* = developmental age (months); *DA coefficient* = 100xDA/chronological age (months)

**Table 2:** Outcomes for children at baseline and after two years of treatment

		Baseline		After two years			
Measures	*n*	M	SD	M	SD	Z	*r*
VABS communication skills DA	15	10	8.2	24.3	16.4	−3.409^***^	0.88
VABS communication skills DA quotient	15	20.6	17.9	37.8	26.1	−3.210^*^	0.83
VABS daily living skills DA	15	17.7	5.1	33.3	11.3	−2.273^***^	0.58
VABS daily living skills DA quotient	15	42.9	11	49.6	14.8	−1.620	0.42
VABS socialisation skills DA	15	9.9	5.6	21.7	12.3	−3.412^*^	0.88
VABS socialisation skills DA quotient	15	22.6	12.2	32.1	16.1	−2.040^*^	0.53
VABS motor skills DA	13	27.4	9.5	41.4	12.6	−3.078^***^	0.85
VABS motor skills DA quotient	13	65.2	18.6	62.5	18.6	0.419	0.12
ADOS-G total score	14	19.14	2.6	17.0	2.9	−1.795	0.48
PSI total percentile rank Mother	20	90.6	9.5	87.4	18.8	−0.181	0.04
PSI total percentile rank Father	18	79.6	27.2	77.3	29.9	−0.666	0.16

*p < .05; **p < .01; ***p < .001

*DA* = developmental age (months); *DA coefficient* = 100xDA/chronological age (months)

As explained above, baseline assessment of IQ was feasible only in six children. Thus, a comparison of IQ-scores became possible only for the assessments made at the one year and the two year follow-up. These data reflected significant gains for the SON-R developmental age total (mean = 40.9, SD = 14.9 vs. mean 51.8, SD = 20.1, z = −3.563, *p* = 0.001, *r* = 0.79) with a large effect size and the SON-R total standard score IQ (Mean = 70.0, SD = 19.4 vs. Mean = 75.5, SD = 24.5, z = −1.915, *p* < 0.05, *r* = 0.43) with a medium effect size.

The changes in autism symptoms as indicated by ADOS scores were not significant after two years of intervention (table 2). In the ADOS-G, only two of the 16 children scored below the autism cut-off of 12 after two years of intervention. Of the 14 children with data at baseline and after two years of intervention, 9 children (64.3%) improved on the total ADOS-G (on average 4.5 points; range 2–9 points). The total ADOS-G score of one child (7.1%) did not change and 4 children (28.6%) showed higher total ADOS-G scores after two years of intervention (on average 2.75 points; range 1–4 points). Furthermore, none of the changes after two years of intervention in the parent PSI scores were significant. The parental stress level stayed high, but did not increase over the entire course of the treatment program (table 2).

## Discussion

Our sample included a high number of children with co-occurring medical conditions and numerous families from outside of Switzerland. This sample composition reflected the fact that due to limited treatment options in Zurich we intended to provide sufficient opportunities also to severely disabled children and/or disadvantageous families including the high proportion of migrants in the city. Whether this sample composition had an impact on the results remains an open question.

In accordance with findings from previous studies [8, 9, 17], the EIBI program that was developed at the CAPS in Zurich, Switzerland, also showed an overall effectiveness within the clinical setting. However, more detailed comparisons across studies should be regarded with caution. For instance, data on IQ were missing in a sizeable proportion of our children because they were not testable at baseline. Thus, no definite conclusion can be drawn regarding the IQ gain during the intervention. It may be noticed that after one year of intervention 20% of the individuals had IQ scores within the average range (>85) which is comparable to the 18% rate of individuals in the intervention group and significantly higher than the 3.3% rate in the waiting list group of the study by Flanagan et al. [17]. Furthermore, the mean IQ score of 72.13 after two years of intervention was comparable to the mean IQ score of 66.6 for the experimental group in the study by Eldevik et al. [8] and the 72.57 IQ score in the study by Grindle et al. [9] and also higher than the score of 52.2 in the comparison group of the study by Eldevik et al. [8]. Thus, these findings parallel to others provide some indirect support but no definite proof that the intervention assisted our children in their cognitive development.

The VABS scores can only be compared to the scores in the study by Flanagan et al. [17]. After intervention, the children in our sample showed markedly lower developmental coefficient scores in the communication domain (30.03 vs. 46.6) and less markedly but still lower scores in the domain socialisation (28.13 vs. 33.90), but very similar scores in the domain daily living skills (46.18 vs. 44.83). At baseline the CAPS scores were very similar to the scores in the study by Flanagan et al. [17] in the domains of communication (21.00 vs. 25.47), socialisation (22.59 vs. 24.08) and daily living skills (41.59 vs. 42.79). Thus, the differences after one year of intervention in the two studies cannot be explained by major differences at baseline and should be regarded with caution. Rather than differences in the effectiveness of the intervention, sample differences may be more relevant.

In contrast to the positive changes in the VABS and the SON-R, the ADOS-G findings reflected only limited changes of the autistic symptoms over time. However, this had to be expected given that the ADOS-G is not an instrument suitable for evaluating changes over time but, rather, for the classification of autism. Furthermore, the PSI findings indicate that the burden of having a child with autism did not decrease significantly for the parents during the intervention.

In our assessment we also asked about the overall effect of the intervention on the family and whether or not the benefits of the intervention were worth the time and effort. This data was analysed in an unpublished master’s thesis for a small number of the treated children after one year of intervention in terms of a pilot study [30]. All the families said that the effect of the intervention on the family was very positive and all but one father said that the benefits were worth the time and effort. Since these responses did not differentiate between varying outcome results, we concluded that there was a response bias and we did not analyse this data quantitatively for all the children.

Although the overall outcome of the intervention in our project was very satisfactory, it was less favourable than the outcome obtained under controlled research conditions (e.g., [4, 31]). In addition, the study methods were different in terms of not having a control condition and there were also further limitations. First, many of the children enrolled in the program were so severely impaired that structured assessments using standardized tests and implementation of the treatment program without modifications and adjustments were not feasible. As a result, there was a sizeable amount of missing data jeopardizing the statistical analyses. For instance, the small sample sizes including non-normal distributions of scores restricted the analyses to multiple non-parametric tests with an inflated risk of type-1 errors. However, the observed significant statistical findings, particularly at the two year follow-up were robust enough to guard against chance findings.

Furthermore, many challenges had to be faced at the CAPS and the intervention model had to be changed numerous times. Applying for financial resources, finding and training new co-therapists regularly and adapting the intervention model to the cultural and political conditions in Switzerland took up a lot of time and resources. These requirements absorbed a substantial amount of professional time and energy, which could have been invested more directly in the intervention for the children under more favourable conditions.

## Conclusions

Even though a lot has been done for a rather small number of children and their families, the early intervention project did not have the kind of impact we had been hoping for in Switzerland. Even the families of the treated children faced many obstacles when the children entered kindergarten. Many of the schools were inexperienced in teaching children with autism and the staff had pronounced resentments against ABA. In some schools, the prejudices against ABA and EIBI were so marked that they did not wish to collaborate with the EIBI staff at all. Many schools were either not open to the idea or did not have the financial resources or legal basis to hire co-therapists to sufficiently assist the students in the classroom. For many of the children the progress they made in early intervention was not enough to function in the classroom without structured and systematic teaching and immediate feedback and the schools were not willing or able to provide these conditions. The gap between the intensive intervention program and the actual school environment was still very large.

In contrast, some schools were very open and eager to learn from the parents and the rest of the child’s treatment team. Thus, these children continued to progress within the school setting and the knowledge about ABA and effective teaching of children with autism was successfully disseminated to the school staff. However, even though a lot has been done in terms of further education of teachers in the last ten years, most schools still are ill - prepared and insufficiently trained to effectively teach autistic children. This conclusion is very much in accordance with the recent critical review by Keenan et al. [19] on the rather unsatisfactory implementation of ABA in Europe.

Because EIBI is mostly not covered by insurance companies or the government, other Swiss centres have not adopted the model. There are very few private service providers serving families with an autistic child in Switzerland because parents have to pay the program themselves. Thus, the opportunities for psychologists and special education teachers with in-depth knowledge of ABA, EIBI and autism are rather limited and most of our more than 200 trained co-therapists are working in other fields now using only a small fraction of their specialized knowledge.

Furthermore, the guidelines defined by the Swiss Federal Social Insurance Office are not meeting the requirements of internationally accepted guidelines in terms of credentials and training of experts supervising an EIBI program as set by the Behavior Analysis Certification Board (BACB) and defined in the practice guidelines for ABA and the treatment of ASD (BACB [32]) designed for funding agencies to set their standards. Also in Switzerland, it would make more sense to set guidelines for supervision according to these international guidelines. Despite of the changes seen in the last ten years, treatment for a child with autism in Switzerland still implies a constant battle on all grounds for the parents. Little has changed in terms of dissemination and availability of ABA and EIBI throughout Switzerland.

For the next few years, the EIBI team of the CAPS will concentrate on evaluating the intervention model with the adaptations that had to be made due to the guidelines set by the Federal Social Insurance Office, thus improving the quality of this intervention model and finding ways to disseminate EIBI. One way will consist of collaborating with qualified independent service providers that have the credentials (BCBA) and provide good quality of EIBI so that families can get the flat rate through the public CAPS to pay for some parts of the services. There is a great need for professionals offering supervision of EIBI programs. The CAPS cannot take all families asking for service and has to refer families to private service providers. Because the CAPS is government-subsidized, the government can determine on the grounds of the budget whether the service for children with autism will have a chance to expand.

However, a reasonable policy should also consider both the indication and the limitation of EIBI. This intervention is intended only for toddlers and preschoolers with ASD and is expensive, time-limited, much more effective for some children than others, and composed of many features that are under-researched and controversial (e.g., appropriate dose, curriculum content, and intervention techniques). Beyond EIBI, many other ABA intervention approaches are available for children and adults with ASD, but details on how to administer most of these approaches are scant [33].

Further funding is another major necessary goal to achieve in order to provide high quality of intervention. If co-therapists can be paid higher salaries and job opportunities are available in early intervention, the motivation to stay on full-time or for a longer period of time is higher. This would lead to less turn-over and more qualified staff.

## Abbreviations

ABA: Applied behaviour analysis
ADOS: Autism Diagnostic Observation Schedule
BCABA: Board Certified assistant Behavior Analyst
BCBA: Board Certified Behavior Analyst
CAPS: Child and Adolescent Psychiatric Service
DA: Developmental age
EIBI: Early intensive behavioural intervention
PECS: Picture exchange communication system
PSI: Parenting Stress Index
SON-R: Snijders-Oomen non-verbal intelligence test
VABS: Vineland Adaptive Behaviour Scale
